# *Epilepsia Open*—August 2026 Announcements

**DOI:** 10.1002/epi4.70308

**Published:** 2026-08-03

## ILAE CONGRESSES

Curso virtual sobre epilepsia en atención primaria para América Latina 2026

3 August–27 September 2026

Online

1º Curso Latinoamericano de EEG Pediátrico

6–9 August 2026

TBC

16th European Epilepsy Congress

5–9 September 2026

Athens, Greece

16th International Summer School for Neuropathology and Epilepsy Surgery

10–13 September 2026

Erlangen, Germany

8th East Mediterranean Epilepsy Congress & 14th Saudi Epilepsy Society Congress

29–31 October 2026

Al Khobar, KSA

16th Asian & Oceanian Epilepsy Congress

19–22 November 2026

Kuala Lumpur, Malaysia

## 2027

16th ILAE School on Pre‐Surgical Evaluation for Epilepsy and Epilepsy Surgery

11–15 January 2027

Brno, Czech Republic

37th International Epilepsy Congress

28 August–1 September 2027

Amsterdam, Netherlands

## WEBINARS

Project ECHO – TUFH 2026 Conference

6 August 2026

Project ECHO – University of Plymouth

15 September 2026

Project ECHO: Epilepsy and Intellectual Development Disabilities – Insight into Tuberous Sclerosis Complex

19 September 2026

Project ECHO – University of Nebraska Medical Center

10 October 2026

Genomics – when, why and how?

ILAE e‐Forum Series 2026

21 October 2026

Neuroimaging – what, why and when

ILAE e‐Forum Series 2026

25 November 2026

## OTHER CONGRESSES

ECON 2026

7–9 August 2026

Ludhiana, India

Epilepsy Imaging in Resource Strained Settings

31 August–12 September 2026

Nigeria, Malaysia, Morocco

Workshop on Combined EEG‐fMRI in epilepsy

9–10 September 2026

Athens, Greece

Himalayan Epilepsy Conference 2026

18–19 September 2026

Kathmandu, Nepal

19th Baltic Sea Summer School on Epilepsy

22 September–6 October 2026

Online

8th PED‐EEG

26–29 September 2026

Bologna, Italy

2026 CLAE Annual Scientific Meeting

2–4 October 2026

Niagara‐on‐the‐Lake, Ontario, Canada

ILAE British Branch 2026 Annual Scientific Conference

8–10 October 2026

Harrogate, UK

XII Congreso de la Sociedad Española de Epilepsia

15–17 October 2026

Córdoba, Spain

Annual Meeting of the German‐Austrian‐Swiss Epilepsy Working Groups 2026

27–30 October 2026

Vienna, Austria

5th Video‐EEG in Paediatric Epilepsies

29–31 October 2026

Madrid, Spain

1st Swiss Neuro Week

18–20 November 2026

Bern, Switzerland

Epilepsy Society of Australia 40th Annual Scientific Meeting

4–6 November 2026

Melbourne, Victoria, Australia

EPIBAQ 2026

12–14 November 2026

Barranquilla, Colombia

ILAE British Branch Clinical Epilepsy Course for Doctors in Training & Allied Healthcare Professionals

21 November 2026

Birmingham, UK

20th Asian Epilepsy Surgery Congress

22–23 November 2026

Kuala Lumpur, Malaysia

43rd International Conference on Advances in Psychiatry and Mental Health

7–8 December 2026

Dubai, United Arab Emirates

Encephalitis 2026

7–8 December 2026

London, UK & Online

## 2027

5th European Congress of Neurology and Neuropsychiatry (NEURO 2027)

22–23 February 2027

Rome, Italy

European Workshop on Developmental and Behavioural Outcome Measures in Developmental and Epileptic Encephalopathies (DEE)

25–27 February 2027

Leuven, Belgium

Fourth International congress on Structural Epilepsy & Symptomatic Seizures

7–9 April 2027

Gothenburg, Sweden

